# Molecular Markers for Thyroid Cancer Diagnosis: Insights from MAPK Pathway Gene Expression Analysis

**DOI:** 10.3390/biomedicines13071577

**Published:** 2025-06-27

**Authors:** Breno Pupin, Ramon Varella Diniz, Maricilia Silva Costa, Maurilio Jose Chagas, André Bandiera de Oliveira Santos, Renata de Azevedo Canevari

**Affiliations:** 1 Laboratório de Genética Molecular—GeneLab, Instituto de Pesquisa e Desenvolvimento, Universidade do Vale do Paraíba—UNIVAP, Avenida Shishima Hifumi 2911, Urbanova, São José dos Campos 12244-000, SP, Brazil; 2 Laboratório de Bioquímica Aplicada à Engenharia Biomédica, Instituto de Pesquisa e Desenvolvimento, Universidade do Vale do Paraíba—UNIVAP, Avenida Shishima Hifumi 2911, Urbanova, São José dos Campos 12244-000, SP, Brazil; 3 Hospital Policlin, Av. Nove de Julho, São José dos Campos 12243-001, SP, Brazil; 4 Instituto do Câncer do Estado de São Paulo, ICESP, Avenida Dr. Arnaldo 251, Cerqueira César 01246-000, SP, Brazil

**Keywords:** thyroid carcinomas, diagnostic, MAPK pathway, gene expression

## Abstract

**Background and Objectives:** Thyroid cancer is the prevailing endocrine malignancy, with incidence growing over the last decades in the world. The current diagnostic techniques often yield inconclusive results, emphasizing the need for more effective diagnostic approaches. Molecular profiling emerges as a promising avenue for carcinoma differentiation, offering precise insights to guide patient selection for surgical intervention. This study aimed to identify molecular markers in thyroid cancer through the expression analysis of genes within the MAPK pathway, aiming to enhance the sensitivity and specificity of carcinoma diagnosis. **Methods:** Through a comparative analysis of malignant and benign thyroid samples, we identified 46 genes of the MAPK pathway that exhibited differential expression by PCR array analysis. **Results:** Validation through RT-qPCR and in silico analysis using TCGA confirmed significant results for CCNA1, CDKN1C, CREB1, FOS, HSPA5, JUN, MAP2K6, and SFN genes identified in our cohort, reinforcing the relevance of these biomarkers. Specifically, noteworthy are our findings regarding the potential diagnostic value of CCNA1 and SFN genes in papillary thyroid carcinoma, while the reduced expression of CDKN1C, FOS, and JUN genes in follicular carcinoma suggests their value in distinguishing the thyroid pathologies. **Conclusions:** This study identifies promising diagnostic markers, namely CCNA1, CDKN1C, FOS, JUN, and SFN genes, which have the potential to enhance clinical decision-making in thyroid cancer.

## 1. Introduction

Thyroid carcinoma is one of the most common malignancies of endocrine organs, with an increased number of cases each year. This malignancy, representing the ninth cancer in incidence, approached 590,000 new cases in 2020, with almost 44,000 deaths attributed to this carcinoma and its complications. This raises concerns about this public health issue [1]. In 2022, the number of total cases was around 821,000, rising to seventh position in the worldwide ranking, and the estimated number of deaths increased to 47,000 [2]. According to the International Agency for Research on Cancer (IARC), this pathology occurs any gender and across a range of ages, is well common in women approaching 75% of diagnostics worldwide and the median age of diagnosis is 50s, even though is the most common cancer in adolescents and adults around 16–33 years old [3].

Papillary thyroid carcinoma (PTC) is the most common carcinoma of the thyroid gland, which approaches 80% of thyroid cancer cases, while follicular thyroid carcinoma (FTC) represents around 15–20% of cases [4]. Although both PTC and FTC are classified as differentiated thyroid carcinomas and generally associated with favorable outcomes, accurate histological classification remains clinically important. PTC typically exhibits an indolent course with excellent long-term survival and a low risk of distant metastasis. In contrast, FTC, while also demonstrating good overall survival, has a greater tendency for hematogenous dissemination, particularly to the lungs and bones, which may lead to worse clinical outcomes in some patients. Therefore, precise diagnosis between these subtypes is critical, as it directly impacts treatment decisions, including the extent of surgery, the need for radioactive iodine (RAI) therapy, and the intensity of follow-up protocols. This clinical demand, combined with the well-known subjectivity and limitations of cytological analysis, highlights the need for alternative methodologies capable of improving the accuracy of thyroid tissue differentiation, diagnosis, and therapeutic decision-making [5]. In this context, the possibility of establishing a sensitive and accurate screening diagnosis method is of utmost importance.

New diagnosis forms and therapies have been developed in the molecular biological guideline [6]. The use of molecular analysis in clinical routine, especially the analysis of specific mutations and gene expression, has increased considerably. Molecular biology techniques have been widely applied to investigate gene expression and understand the complex interactions among biochemical pathways that contribute to cancer development and progression. Currently, the World Health Organization (WHO) recognizes the importance of these methodologies and prescribes them in the categories of papillary and follicular carcinomas [1,2].

Some signaling pathways play important roles in thyroid function, and many associated genes are abnormally expressed in patients with thyroid cancer [7,8,9]. The mitogen-activated protein kinase/extracellular signal-regulated (MAPK/ERK or MAPK) pathway plays an important role in the control of many cellular functions, including cellular interactions, migration, proliferation, differentiation, apoptosis, protein biosynthesis, gene transcription, and metabolism rate, among others [10,11]. Alterations in the expression of genes involved in the activation of the MAPK pathway can contribute to tumor development. Therefore, detecting these molecular changes offers potential for more precise and sensitive cancer diagnostic strategies [12]. The importance of this pathway has been well established in thyroid cancer [11,12], where in PTCs the MAPK pathway is driven by the activation of point mutations of the BRAF and RAS genes and in the RET/PTC rearrangement, these genetic alterations being found in more than 70% of PTCs [13]. Already, in FTC, the most frequent alterations include mutations of the RAS gene, in addition to the PAX8-PPARγ rearrangement [14]. Many of these mutations, particularly those that lead to the activation of the MAPK pathway, are being studied intensively as diagnostic targets for thyroid cancer [11,15,16,17,18,19].

The aim of this study was to identify molecular markers indicative of thyroid cancer through expression analysis of genes within the MAPK pathway, evaluating their potential for diagnostic prediction of these lesions. Initially, Polymerase Chain Reaction array (PCR array) gene expression was conducted on 24 thyroid samples, revealing a 46-gene set linked with diagnosis. Subsequently, Reverse Transcription Quantitative PCR (RT-qPCR) was employed to validate the PCR array findings on a panel of candidate marker genes in a set of 40 cases, comprising the initial 24 samples previously analyzed by RT-qPCR as well as an additional 16 samples. The objective was to discern thyroid tissues accurately, thus augmenting the sensitivity and specificity of carcinoma diagnosis.

## 2. Materials and Methods

### 2.1. Ethical Aspects

This study was approved by the Research Ethics Committee of the University of Vale do Paraíba (Univap) (n° 1.806.781). Tissue samples were collected from hospitals in São José dos Campos, São Paulo, and from the Department of Head and Neck Surgery at the Clinical Hospital, School of Medicine, University of São Paulo (USP), Brazil. All participants received detailed information about the study and provided written informed consent prior to sample collection.

### 2.2. Samples

A total of 40 fresh tissue specimens were previously collected through surgical excisional biopsies obtained from total thyroidectomy. All samples were histopathologically analyzed according to the diagnostic criteria of the Brazilian Society of Pathology (BSP) for thyroid cancer. Immediately after collection, the specimens were frozen in liquid nitrogen and stored in cryogenic tubes. Each sample was divided into two portions: one for histopathological evaluation and the other stored at –80 °C for PCR array and RT-qPCR analyses. Macrodissection was performed using a scalpel to ensure that the selected fragments contained at least 80% tumor cells.

The PCR array analysis was conducted on a total of 24 samples, comprising 12 benign tissue samples, which included six samples of goiter and six samples of follicular adenoma and follicular hyperplasia, as well as 12 malignant tumor samples consisting of six PTC samples and six FTC samples. For the RT-qPCR analysis, a total of 40 samples were assessed, which included samples previously analyzed by PCR array and additional samples. These samples were histologically classified as follows: normal tissue (7 samples), which also served as a reference for data normalization; adenomatous goiter (6 samples); adenoma and hyperplasia follicular (9 samples); PTC (10 samples); and FTC (8 samples).

### 2.3. Design

For gene expression analysis using PCR array and RT-qPCR, the samples were categorized into five groups: group 1 comprised benign tissues (including normal tissue, goiter, follicular adenoma, and follicular hyperplasia), group 2 included malignant tissues (PTC and FTC), group 3 consisted of goiter samples, group 4 contained PTC samples, and group 5 encompassed FTC samples. Analysis 1 compared group 2 to group 1, Analysis 2 compared group 3 to group 1, Analysis 3 compared group 4 to group 1, Analysis 4 compared group 5 to group 1, and Analysis 5 compared group 5 to group 4.

### 2.4. Extraction of RNA and Reverse Transcription

Total RNA samples were isolated using silica membrane from a RNeasy Mini-Kit (Qiagen, Hilden, Germany). All samples were treated with DNase I and RNeasy MinElute Cleanup Kit (Qiagen, Hilden, Germany). Total RNA quantification was obtained using the Nanodrop device, where purity and integrity were confirmed by ultraviolet absorption spectroscopy (A260/A280 and 260/230 ratio), and 1 μg of RNA per sample was used for cDNA synthesis. Reverse transcription reactions were performed using an RT^2^ First Strand Kit (Qiagen, Hilden, Germany) according to the manufacturer’s protocol and conducted for 5 min at 42 °C, 15 min at 42 °C, and the reaction mixture was subsequently inactivated for 5 min at 95 °C.

### 2.5. RT^2^ Profiler PCR Array

Gene expression analysis by PCR array was performed using the ABI Prism 7500 Sequence Detection System (Life Technologies, Carlsbad, CA, USA). Reactions were carried out with the RT2 SYBR Green qPCR Master Mix and the Human MAP Kinase Signaling Pathway RT^2^ Profiler PCR Array (PAHS-061Z; Qiagen, Hilden, Germany), that contains primer assays for 84 representative genes from MAP Kinase pathway, five housekeeping genes that enable normalization of data (ACTB, B2M, GAPDH, HPRT1 and RPLP0), a control genomic DNA (GDC), three reverse-transcription controls (RTC), and three positive PCR controls (PPC). The GDC control is designed to detect potential genomic DNA contamination with a high level of sensitivity. The RTC control evaluates the effectiveness of the reverse transcription reaction by amplifying a template generated from an external RNA control included in the RT2 First Strand Kit. The PPC consists of a synthetic DNA sequence and its corresponding detection assay, serving to verify the efficiency of the PCR amplification process. All controls were supplied in replicates, allowing assessment of inter-well and intra-plate consistency.

Equal aliquots of cDNA and RT^2^SYBR Green qPCR Master Mix (25 μL) were added to each well of the same PCR Array plate containing the predispensed gene-specific primer sets. The reaction conditions were as follows: 60 °C for 1 min, followed by 40 cycles at 95 °C for 15 s, 55 °C for 40 s, and 72 °C for 30 s. Following the PCR array, the data from all samples were processed with the same Ct threshold, which was assigned an arbitrary value of 0.067761 based on the average across all plates and manual adjustment for optimal fit. The baseline was established at three cycles below the lowest observed Ct. The data with the threshold values were exported and analyzed by web-based RT^2^ Profiler PCR Array Data Analysis template v 3.5 (https://geneglobe.qiagen.com/us/analyze, accessed in 17 January 2022). 

Statistical analysis of real-time PCR was carried out using the unpaired Mann–Whitney test, with *p*-values below 0.05 considered statistically significant. Fold changes were calculated in the PCR Array Data Analysis template v3.3 (SABiosciences, Frederick, MD, USA, accessed in 17 January 2022). A fold-change value greater than 2 was considered indicative of gene overexpression, while a fold-change value less than 0.5 was defined as downregulation.

### 2.6. Evaluation of Transcript Expression by RT-qPCR

To validate the diagnostic potential of determined genes identified as candidates for molecular markers through PCR array analysis, a validation analysis was conducted using RT-qPCR (Table 1). This validation focused on 10 selected genes (CCNA1, CDKN1C, CREB1, FOS, HSPA5, JUN, KSR1, MAP2K6, MAPK8IP2, and SFN), previously identified through the PCR array technique.

RT-qPCR reactions for 10 genes and the reference gene (MRLP19) were performed in duplicate using the ABI Prism 7000 Sequence Detection System (Thermo Fisher Scientific, Inc., Waltham, MA, USA). The reactions utilized Platinum^®^ SYBR^®^ Green qPCR SuperMix-UDG (Applied Biosystems, Waltham, MA, USA; Thermo Fisher Scientific, Inc.) in a total volume of 10 µL, following the manufacturer’s instructions. The reverse transcription step consisted of an initial cycle at 95 °C for 10 min, followed by 40 cycles of 15 s at 95 °C and 1 min at 60 °C. A dissociation curve was included in all experiments.

Primer Express software (v3.0; Applied Biosystems; Thermo Fisher Scientific, Inc., Foster City, CA, USA) was used to design the primers. The sequences and additional details of the gene-specific RT-qPCR assays are provided in Table 1. To prevent amplification of contaminating genomic DNA, primers were designed at exon–exon junctions or located in different exons.

For the 10 genes selected for validation by RT-qPCR, as well as for the endogenous control (MRPL19), the primer amplification efficiency was determined, and the appropriate sample dilution was obtained. Amplification curves for all genes were generated using serial dilutions of cDNA from healthy thyroid and thyroid carcinoma samples (100, 20, 4, 0.8, and 0.16 ng/µL). The standard curves for both target and reference genes showed comparable amplification efficiencies exceeding 90%. Quantitative data analyses were performed with the Sequence Detection System software (v1.0; Applied Biosystems; Thermo Fisher Scientific, Inc., Foster City, CA, USA).

Gene expression levels were evaluated by the relative ΔΔCt method, where the average Ct of each target gene per sample was normalized against the average Ct of the reference gene. The MRLP19 gene was chosen as the endogenous control due to its ability to enhance accuracy and sensitivity in detecting smaller expression changes. Expression levels of target genes in tumor samples were calculated as fold changes relative to a pool of non-tumor control tissues using the formula: 2^(∆Ct test sample − ∆Ct control sample)^. Statistical analysis of gene expression data was performed by comparing the median 2^ΔΔCt^ values between groups using the GraphPad Prism software (version 5.00; GraphPad Software, San Diego, CA, USA). The Mann–Whitney test was used to ascertain the statistical significance of the expression levels evaluated by RT-qPCR and considered statistically significant with *p*-values < 0.05.

Gene expression validation was performed using publicly available RNA-seq data from The Cancer Genome Atlas (TCGA), accessed via the UALCAN portal (https://ualcan.path.uab.edu/analysis.html, accessed in 20 June 2025).

## 3. Results

**Figure 1:**
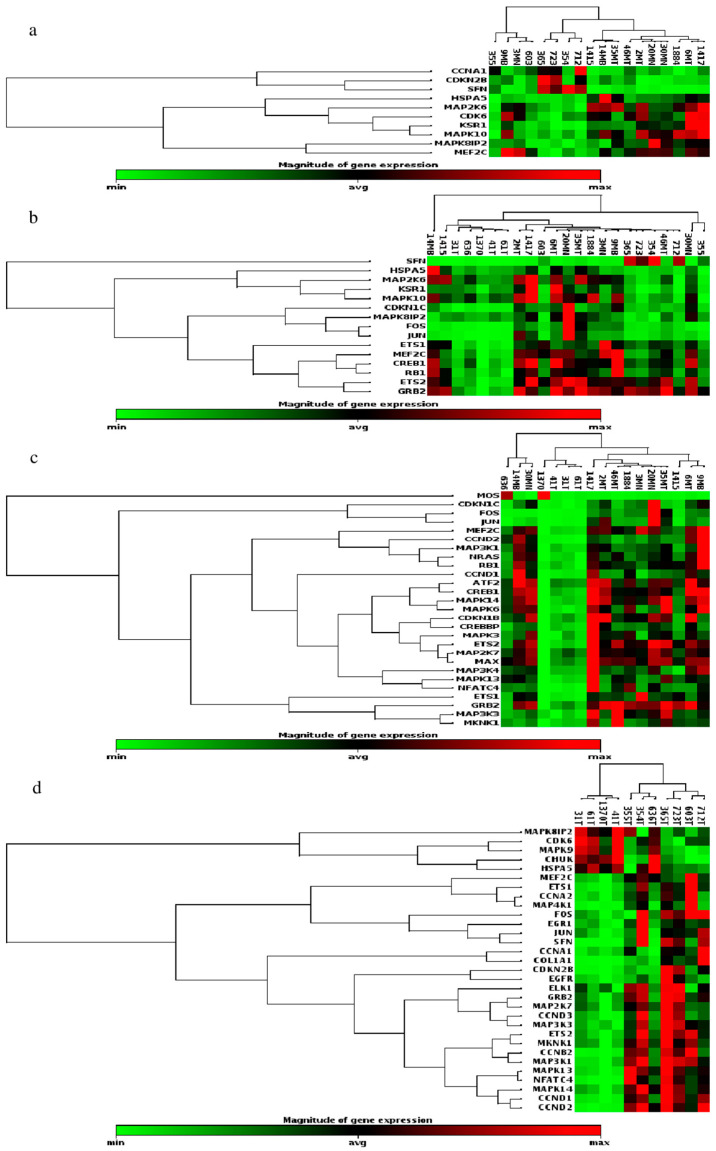
Differential expression of MAPK pathway-related genes in thyroid lesion samples analyzed by PCR array. Out of the 84 genes examined, 46 genes showed significant differential expression (fold change ≥ 2 for upregulation or ≤0.5 for downregulation, with *p* < 0.05). (**a**) Analysis 1: malignant tumors (PTC and FTC) (group 2) vs. normal thyroid and benign tumors samples (group 1); (**b**) Analysis 3: PTC samples (group 4) vs. group 1; (**c**) Analysis 4: FTC samples (group 5) vs. group 1; (**d**) Analysis 5: FTC (group 5) vs. PTC samples (group 4). Bars represent the average fold change, with min (minimum) and max (maximum) values indicating variability among samples. The exact fold-change values and statistical details for each gene are provided in Supplementary Table S1.

Five analyses comparing the different groups were conducted. Taking into account all the analyses performed, out of the 84 MAP Kinase Pathway genes examined, 46 genes displayed significant differential expression (Supplementary Table S1, Figure 1). In Analysis 1, which compared group 2 (samples from PTC and FTC) to group 1 (samples from normal thyroid tissues and benign tumors), 15 genes exhibited significant differential expression between the groups. Among these, 14 genes (CDKN1C, CREB1, ETS1, ETS2, FOS, GRB2, HSPA5, JUN, KSR1, MAPK10, MAPK8IP2, MAP2K6, MEF2C, and RB1) decreased expression, while the SFN gene demonstrated increased expression in group 2 compared to group 1 (Figure 1a). Notably, Analysis 2 revealed no significant differences in gene expression between goiter samples (group 3) and normal and benign tissues (group 1).

Significant differential expression was observed in 10 genes when comparing PTC samples (group 4) with group 1 (Analysis 3). CDK6, HSPA5, KSR1, MAPK10, MAPK8IP2, MAP2K6, and MEF2C genes exhibited decreased expression, while three genes (CCNA1, CDKN2B, and SFN) demonstrated increased expression in PTC samples compared to normal and benign tissues (group 1) (Figure 1b). In Analysis 4, twenty-seven genes exhibited significant differential expression between FTC samples (group 5) and benign tumor samples (group 1). Among these, 26 genes (ATF2, CCND1, CCND2, CDKN1B, CDKN1C, CREBBP, CREB1, ETS1, ETS2, FOS, GRB2, JUN, MAPK3, MAP2K6, MAPK13, MAPK14, MAP2K7, MAP3K1, MAP3K3, MAP3K4, MAX, MEF2C, MKNK1, NFATC4, NRAS and RB1) displayed decreased expression, while one gene (MOS) exhibited increased expression in FTC samples compared to group 1 (Figure 1c). In Analysis 5, which compared group 5 to group 4, 31 genes displayed significant differential expression in the comparison between the FTC samples (group 5) and PTC samples (group 4). Among these, 26 genes (CCNA1, CCNA2, CCNB2, CCND1, CCND2, CCND3, CDKN2B, COL1A1, EGFR, EGR1, ELK1, ETS1, ETS2, FOS, GRB2, JUN, MAPK13, MAPK14, MAP2K7, MAP3K1, MAP3K3, MAP4K1, MEF2C, MKNK1, NFATC4, and SFN) exhibited reduced expression, while five genes (CDK6, CHUK, HSPA5, MAPK8IP2, and MAPK9) showed increased expression in FTC compared to PTC samples (Figure 1d) (*p* ≤ 0.05).

The RTC and PPC controls indicated that there was no contamination in the RNAs of the samples and no impurity in the RNA sample that affected the PCR amplification, respectively. In addition, consistency and high reproducibility were verified between the different PCR array plates analyzed, demonstrated by the low variation between the means of the replicates of the RTC and PPC controls. GDC showed the absence of contamination by genomic DNA.

Among the genes displaying differential expression, it was observed that the MEF2C gene exhibited differential expression in all four analyses, while eight genes (ETS1, ETS2, FOS, GRB2, HSPA5, JUN, MAPK8IP2, and SFN) showed differential expression in three analyses. Additionally, 19 genes (CCNA1, CCND1, CCND2, CDKN1B, CDKN1C, CDKN2B, CDK6, CREB1, KSR1, MAPK10, MAPK13, MAPK14, MAP2K6, MAP2K7, MAP3K1, MAP3K3, MKNK1, NFATC4, and RB1) exhibited differential expression in two analyses, while 18 genes (ATF2, CCNA2, CCNB2, CCND3, CHUK, COL1A1, CREBBP, EGFR, EGR1, ELK1, MAPK3, MAPK6, MAPK9, MAP3K4, MAP4K1, MAX, MOS, and NRAS) showed differential expression in just one analysis.

**Figure 2:**
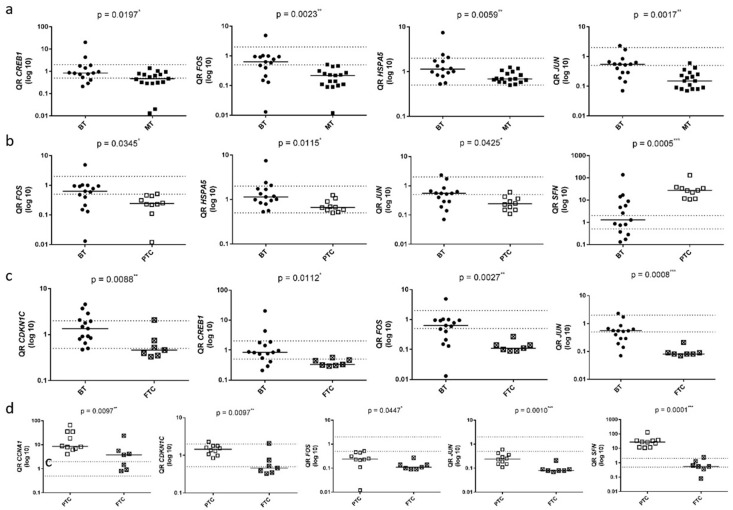
Expression analysis of candidate diagnostic markers in thyroid cancer. The relative expression levels of CCNA1, CDKN1C, CREB1, FOS, HSPA5, JUN, KSR1, MAP2K6, MAPK8IP2, and SFN were determined by quantitative RT-qPCR in thyroid samples. The genes displaying differential expression are shown. (**a**) Analysis 1: MT (group 2) vs. BT samples (group 1); Analysis 3: (**b**) PTC (group 4) vs. BT samples (group 1); (**c**) Analysis 4: FTC (group 5) vs. BT samples (group 1); Analysis 5: (**d**) FTC (group 4) vs. PTC samples (group 5). Each gene was analyzed in duplicate, and expression levels were normalized to the reference gene MRLP19 to ensure consistency across all samples. Relative quantification (RQ) was used to represent gene expression levels. BT: normal thyroid and benign tumor samples, MT: malignant tumors (PTC and FTC), PTC: papillary thyroid carcinoma, and FTC: follicular thyroid carcinoma. Median gene expression levels for each sample group are presented. All genes showed statistically significant differential expression, with *p* < 0.05, as determined by the Mann–Whitney test: * *p* < 0.05, ** *p* < 0.01, *** *p* < 0.001.

Due to their significant differential expression in at least three out of the five conducted analyses and their key roles within the MAPK molecular pathway, the genes CCNA1, CDKN1C, CREB1, FOS, HSPA5, JUN, KSR1, MAP2K6, MAPK8IP2, and SFN were selected for validation analysis using RT-qPCR. Consistent with the PCR array data, these genes, with the exception of KSR1 and MAPK8IP2, exhibited differential expression in at least one of the analyses when the same and additional samples (40-sample set) were assessed by RT-qPCR (Figure 2 and Figure S1).

Figure 2a illustrates the comparison between carcinoma samples (PTC and FTC) (group 2) and normal and benign samples (group 1). In this comparison, the CREB1, FOS, HSPA5, and JUN genes exhibited a significant downregulation in malignant tumors compared to group 1 (*p* < 0.05) (Analysis 1). Similarly, to the results obtained from the PCR Array analysis (Analysis 2), significant differential expression was not observed in the comparison of adenomatous goiter samples (group 3) with samples of normal tissue and benign tumors (group 1), except adenomatous goiter as assessed by RT-qPCR.

Figure 2b illustrates the comparison between PTC samples (group 4) and normal and benign samples (group 1). In this comparison, the CCNA1, CREB1, FOS, HSPA5, JUN, MAP2K6, and SFN genes showed significant differences among the groups (*p* < 0.05). The FOS, HSPA5, and JUN genes displayed decreased expression, while the SFN and CCNA1 genes exhibited increased expression in the PTC group compared to group 1. Decreased expression of CREB1 and MAP2K6 was also observed in group 4 relative to group 1 (Analysis 3).

Figure 2c compares the groups of FTC samples (group 5) to normal and benign samples (group 1). In this analysis, the CDKN1C, CREB1, FOS, HSPA5, and JUN genes exhibited significant differential expression, showing reduced expression in the FTC group compared to group 1 (*p* < 0.05) (Analysis 4).

Figure 2d compares the FTC samples (group 5) and the PTC samples (group 4). In this analysis, the CCNA1, CDKN1C, FOS, JUN, and SFN genes exhibited a significant difference (*p* < 0.05), with reduced gene expression observed in the FTC group (Analysis 5).

### TCGA Dataset Analysis

To complement and validate the RT-qPCR results, gene expression data from the TCGA dataset were analyzed for the same gene set in PTC and FTC in comparison with normal and benign thyroid tissues.

In the comparison between PTC and benign thyroid samples, SFN and CCNA1 were significantly upregulated, whereas FOS, HSPA5, JUN, CREB1, and MAP2K6 exhibited significant downregulation, supporting the findings obtained from the RT-qPCR analysis.

Similarly, in the FTC versus normal and benign tissue comparison, the genes CDKN1C, CREB1, FOS, HSPA5, and JUN were also significantly downregulated, corroborating the trends observed in our experimental dataset.

Collectively, these TCGA-based data reinforce the robustness of our RT-qPCR results and underscore the relevance of these genes as potential molecular markers for malignant transformation in thyroid neoplasms, particularly within the MAPK signaling pathway.

## 4. Discussion

Molecular diagnosis, involving the analysis of genes and specific signaling pathways in thyroid cancer, has been increasingly incorporated into clinical practice due to its numerous advantages over histopathological analysis, including greater sensitivity and specificity in distinguishing between benign lesions, malignant tumors, and normal tissue. Given the rising incidence of thyroid lesions, particularly in PTC, it is crucial to develop more precise diagnostic methods that reduce unnecessary surgical procedures or delayed treatment.

The MAPK molecular pathway has been extensively studied for expressing regulatory genes of important functions in carcinogenesis [12]. However, its precise role in thyroid cancer remains elusive. For instance, while mutation analysis of BRAF, RET, and RAS genes, which are part of the MAPK pathway, is already integrated into clinical practice for diagnostic and prognostic purposes in thyroid cancer [20], there is limited understanding of how genes within this pathway contribute to tumorigenesis in these tumors.

This study conducted a comparative analysis between malignant and benign thyroid samples, identifying 46 genes exhibiting differential expression associated with the MAPK pathway through the PCR array technique. Further validation through RT-qPCR on a selected subset of 10 genes confirmed significant results for CCNA1, CDKN1C, CREB1, FOS, HSPA5, JUN, MAP2K6, and SFN genes. The differential gene expression observed across diverse thyroid tissues suggests their direct implication in the carcinogenic processes underlying this pathology, indicating promising diagnostic potential for clinical application. Moreover, the regulatory functions of these genes in various cellular and metabolic processes, including cell proliferation, growth factors, survival mechanisms, motility, metabolic regulation, apoptosis, transcription, and translation [21], emphasize the critical need for molecular investigations into key genes within the MAPK pathway.

The increased expression observed for the CCNA1 gene in the PTC group compared to the group of normal and benign samples, and its reduced expression in the FTC group when compared to the PTC samples, in both the PCR Array and RT-qPCR analyses, suggests this gene as a potential diagnostic marker in PTC. CCNA1 (cyclin A1) is a protein-coding gene belonging to the highly conserved cyclin family, known for its significant role in cell cycle regulation, particularly in germline meiotic events and to a lesser extent in mitotic events. Elevated expression of the CCNA1 gene and protein has been reported in various tumor conditions, such as PTC [21] when associated with pituitary homeobox 2 (PITX2) transcriptional factor, breast cancer [22] when associated with Six1 homeoprotein, prostate cancer [23], leukemic myeloid [24] and ovarian cancer [25].

The assessment of diagnostic potential through gene and protein expression analysis of the CCNA1 gene in thyroid lesions was recently published in a previous study by our group [26]. In this study, the observed increased expression of the CCNA1 gene in the PTC group compared to other thyroid tissues supports its involvement in tumor progression and highlights its potential as a diagnostic marker for PTC. Although it was observed that Cyclin A1 protein levels by IHC analysis were higher in the PTC group, this difference was not statistically significant, likely due to the limited sample size. Nevertheless, this previous investigation underscores the greater sensitivity of gene expression analysis and highlights its importance as a diagnostic marker compared to protein analysis.

The reduced expression of the CDKN1C gene in carcinoma samples (PTC and FTC) (group 2) and FTC samples (group 5) compared to normal and benign samples (group 1) in both the PCR Array and RT-qPCR analyses, as well as in FTC samples compared to PTC group samples in the RT-qPCR analysis, indicates the diagnostic potential of this gene in differentiating FTC samples from other thyroid pathologies. The CDKN1C (cyclin-dependent kinase inhibitor 1C) gene, also referred to as p57KIP2, is part of the Cip/Kip (CDK-interacting protein/protein kinase inhibitory) family. It functions as a negative regulator of cell proliferation, thereby inhibiting cell cycle progression from G1 to the S phase. Some studies have shown that reduced expression of CDKN1C plays a role in tumor progression, supporting its role as a tumor suppressor [27]. Furthermore, a previous study conducted by our group identified differential expression of the CDKN1C gene in primary tumors that exhibited lymph node metastasis compared to those that did not show metastasis [28].

The notable decrease in expression of the FOS and JUN genes, observed in both the malignant samples group (PTC and FTC) (group 2) and the FTC samples group (group 5), when compared to the group of normal and benign samples (group 1), as well as with the reduced expression of these genes detected in the FTC sample group compared to the PTC samples (group 4), in both PCR array and RT-qPCR analyses, supports the hypothesis that both genes emerge as potential diagnostic molecular markers in thyroid cancer, particularly in FTC. FOS (Fos proto-oncogene, AP-1 transcription factor subunit) and JUN (Jun proto-oncogene, AP-1 transcription factor subunit) genes are proto-oncogenes that encode the transcription factor of activator protein-1 (AP-1), protein complex that is a major component in the MAPK signaling pathway [29] and are associated with the development of various tumor types. The FOS gene is involved in multiple tumorigenic processes such as cell motility, proliferation, tumor growth, metastasis, and angiogenesis [30]. Krishna et al. [31] identified decreased expression of the C-FOS gene in patients with oral squamous cell carcinoma (OSCC), suggesting a role in tumor development. This was determined by comparing OSCC with normal oral mucosa samples, revealing reduced gene expression in the OSCC group. Similarly, the JUN gene, the first proto-oncogene identified, plays a crucial role in regulating cell proliferation and carcinogenesis, much like FOS. Chen et al. [29] reported that JUN is involved in extracellular signal transduction processes in PTC, interacting with various transcription factors beyond its typical cofactor FOS. Furthermore, Fry and Inoue [32] described that the AP-1 transcription factor encoded by JUN directly regulates the expression of an essential checkpoint protein during the G1/S phase of the cell cycle, positively influencing this process.

A previous study conducted by our group [33] hypothesized that the decreased expression of the FOS and JUN genes observed in malignant thyroid tumors could lead to a failure in cellular proliferation control, such as the AP-1 transcription complex, which may be correlated with genes responsible for processes like apoptosis and cellular repair. The reduced expression of this gene could consequently favor different carcinogenic processes. Our current study’s findings are consistent with our previous research [33], further substantiating that the FOS and JUN genes serve as potential molecular markers for diagnosing thyroid malignancies, particularly follicular thyroid carcinoma (FTC).

The CREB1 gene exhibited decreased expression in both the malignant sample group (PTC and FTC) (group 2) and the FTC sample group (group 5) compared to the group of normal and benign samples (group 1) through PCR array validated by RT-qPCR analysis, indicating its potential as a molecular diagnostic marker for distinguishing between benign and malignant neoplasms. CREB1 (cAMP response element-binding protein 1) is a widely recognized transcription factor that belongs to the basic leucine zipper (bZIP) family of proteins [34] and has been extensively investigated in various tumors due to its significant impact on cancer development. Previous studies have highlighted CREB1’s involvement in colorectal cancer [35], breast cancer [36], and thyroid cancer [37]. Ref. [38] examined CREB1 expression levels in thyroid cancer cell lines and observed an elevation compared to normal thyroid cell lines. These authors also identified that CREB1 acts as a downstream effector of the MEX3A gene, highlighting its involvement in the regulatory pathway through which MEX3A influences thyroid cancer progression.

In our study, the reduced expression observed for the HSPA5 gene in the malignant tumor samples group (PTC and FTC) (group 2) and in the PTC samples group (group 4), compared to the group of normal and benign samples (group 1) by PCR Array analysis, confirmed by RT-qPCR analysis, indicates this gene as a diagnostic marker in malignant tumors. The HSPA5 (heat shock protein family A member 5) gene codes for an endoplasmic reticulum (ER) chaperone protein, known as binding immunoglobulin protein (BiP), which plays a vital role in regulating ER functions. Consequently, changes in its expression, activation, or suppression have been linked to cancer development [39]. Previous studies in the literature, as well as our results, have detected reduced expression of the HSPA5 gene in thyroid cancer samples compared to non-neoplastic gland tissues [40]. Additionally, low expression of this gene has also been associated with worse tumor staging and cervical metastasis, among other clinicopathological characteristics, with poor prognosis in these tumors [41].

The reduced expression of the MAP2K6 gene in the malignant sample group (group 2) and in the PTC samples group (group 4) compared to the group of normal and benign samples (group 1) suggests that this gene could serve as a diagnostic marker in malignant neoplasms. To our knowledge, no existing studies in the literature have evaluated the expression of this gene in PTC. However, alterations in its expression and its potential as a diagnostic/prognostic marker have been observed in other cancers such as lung cancer [41] and breast cancer [42].

The increased expression observed for the SFN gene in the malignant tumor samples group (PTC and FTC) (group 2) and in the PTC group (group 4) compared to the group of normal and benign samples (group 1), and its reduced expression in the FTC group (group 5) when compared to the PTC samples, in both the PCR Array and RT-qPCR analyses, indicated this gene as a promising diagnostic marker for PTC. SFN (stratifin) encodes a cell cycle checkpoint protein that is associated with translation and initiation factors and functions as a regulator of mitotic translation [43]. This protein plays a key role in preventing DNA errors during mitosis in response to DNA damage. Previous studies have suggested that SFN is involved in various types of tumors, including lung [44], renal [45], liver [46], and breast [47] cancer. In thyroid cancer, as observed in our study, Prasad et al. [48] observed elevated expression of both the gene and its encoded protein in malignant tumors compared to benign thyroid tissues.

Despite the promising results obtained in our study, some limitations should be considered. Although we identified significant gene expression differences between malignant and benign thyroid lesions, we did not assess whether these changes correlate with protein levels, for example, through immunohistochemistry or Western blot analyses. Functional studies, such as gene knockdown or overexpression, were also not performed. However, our aim was to identify gene expression signatures with diagnostic potential, similar to current molecular tests used in clinical practice, such as gene expression-based tests for indeterminate thyroid nodules. Future studies are needed to validate these findings at the protein level and to explore the functional roles of these genes in thyroid tumorigenesis.

## 5. Conclusions

This study provides valuable insights into the molecular landscape of thyroid cancer, particularly focusing on the MAPK pathway. The differential gene expression of CCNA1, CDKN1C, CREB1, FOS, HSPA5, JUN, MAP2K6, and SFN genes across various thyroid tissue types suggests not only their involvement in thyroid carcinogenesis but also their potential diagnostic value. Notably, CCNA1 was significantly upregulated in PTC samples, supporting its role as a diagnostic marker and aligning with its established association with other cancers. Conversely, CDKN1C expression was reduced in carcinoma samples, particularly FTC, indicating its potential utility in distinguishing FTC from other thyroid pathologies, consistent with its role as a tumor suppressor. Moreover, FOS and JUN expression alterations in FTC, along with SFN upregulation in PTC, further highlight these genes as promising diagnostic candidates for specific thyroid cancer subtypes.

Our findings were further validated through in silico analysis using TCGA data, which confirmed the differential expression patterns observed in our cohort, thus reinforcing the potential relevance of these biomarkers. This integrative approach strengthens the robustness of our results and underscores the potential clinical relevance of these molecular markers for thyroid cancer diagnosis.

Overall, our study emphasizes the importance of molecular investigations in comprehending the complexities of thyroid tumorigenesis and in supporting the development of more accurate diagnostic tools, with promising potential for noninvasive screening and clinical use. However, further studies with a larger number of samples are needed to prove the diagnostic utility of these markers and assess their impact on patient outcomes.

## Figures and Tables

**Table 1:** Evaluated gene, chromosome location, amplicon size, and primer sequences used in real-time RT-qPCR.

Gene	Chromosome	Amplicon	Primer
CCNA1	13q13.3	62 pb	5′-ACAGCTGCTCGGTCAGAGA-3′ 5′-TGTGCCGGTGTCTACTTCAT-3′
CDKN1C	11p15.4	65 pb	5′-GGCCTCTGATCTCCGATTTC-3′ 5′-ATCGCCCGACGACTTCT-3′
CREB1	2q33.3	58 pb	5′-GGGCCTGCAAACATTAACC-3′ 5′-AATGGTAGTACCCGGCTGAGT-3′
FOS	14q24.3	60 pb	5′-AGACCGAGATTGCCAACCT-3′ 5′-GAGCTGCCAGGATGAACTCT-3′
HSPA5	9q33.3	57 pb	5′-AACCATCCCGTGGCATAA-3′ 5′- CCTGGACAGCAGCACCATA-3′
JUN	1p32.1	61 pb	5′-AGCGGACCTTATGGCTACAG-3′ 5′-CCAGGTTCAGGGTCATGCT-3′
KSR1	17q11.2	55 pb	5′-AGCCGAACCCCATTTTG-3′ 5′-AGGGTGCTCCTTCTTTGTCA-3′
MAP2K6	17q24.3	59 pb	5′-CACCTTTTATGGCGCACTGT-3′ 5′-CCATGAGCTCCATGCAGATC-3′
MAPK8IP2	22q13.33	63 pb	5′-CAGCCCTGACCTCACTTTCT-3′ 5′-GACCGAGATGTGCTGTTGAC-3′
SFN	1p36.11	72 pb	5′-CATGGAGAGAGCCAGTCTGAT-3′ 5′-GCTGCCATGTCCTCATAGC-3′
MRPL19	2p11.1-q11.1	70 pb	5′-CAGAGATCAGGAAGAGGACTTGGA-3′ 5′-TCTCGACACCTTGTCCTTCGA-3′

## Data Availability

The original contributions presented in this study are included in the article/Supplementary Materials. Further inquiries can be directed to the corresponding author.

## Supplementary Materials

The following supporting information can be downloaded at https://www.mdpi.com/article/10.3390/biomedicines13071577/s1. Table S1: Fold change, fold regulation, and *p*-value values of 46 genes from the MAPK pathway with significant differential expression of thyroid tissue groups using the RT^2^ Profiler PCR Array Data Analysis. Figure S1. Comparison between the means of expression levels by RT-qPCR of all significantly differentially expressed genes in the studied thyroid samples.
