# Supplementary material for: Molecular Markers for Thyroid Cancer Diagnosis: Insights from MAPK Pathway Gene Expression Analysis
Source: Biomedicines. 2025 Jun 27;13(7):1577. doi: 10.3390/biomedicines13071577 (PMC12292540; doi:10.3390/biomedicines13071577)
Supplement: Supplementary file 1 [file biomedicines-13-01577-s001.zip › biomedicines-3696393-supplementary.pdf]

Supplementary Table S1. Fold change, fold regulation and p-value values of 46 genes from the MAPK pathway with significant differential expression of thyroid tissue groups using the RT² Profiler PCR Array Data Analysis.

Position	Gene	Analysis 1 BT with MT			Analysis 2 BT with G			Analysis 3 BT with PTC			Analysis 4 BT with FTC			Analysis 5 PTC with FTC		
		Fold Change	Fold Regulation	p-value	Fold Change	Fold Regulation	p-value	Fold Change	Fold Regulation	p-value	Fold Change	Fold Regulation	p-value	Fold Change	Fold Regulation	p-value
A02	*ATF2*	0.5038	-1.985	0.00001	1.1374	1.1374	0.442834	0.5276	-1.8952	0.000538	0.4766	-2.0983	0.00088	0.9032	-1.1071	0.653111
A04	*CCNA1*	1.925	1.925	0.049778	1.5641	1.5641	0.512958	3.963	3.963	0.000608	0.8094	-1.2355	0.387704	0.2042	-4.8964	0.011704
A05	*CCNA2*	0.7083	-1.4119	0.322617	1.0863	1.0863	0.547746	1.1448	1.1448	0.727504	0.3981	-2.5121	0.276778	0.3477	-2.8757	0.009815
A07	*CCNB2*	0.6441	-1.5526	0.454425	0.8977	-1.1139	0.498303	1.4566	1.4566	0.813692	0.2419	-4.1336	0.159817	0.1661	-6.0211	0.000464
A08	*CCND1*	0.6702	-1.492	0.685647	1.3747	1.3747	0.442754	1.6404	1.6404	0.117088	0.229	-4.3676	0.01287	0.1396	-7.1646	0.000106
A09	*CCND2*	0.6531	-1.5313	0.684318	1.7674	1.7674	0.043726	1.5465	1.5465	0.17479	0.2321	-4.3086	0.030204	0.1501	-6.6632	0.001037
A10	*CCND3*	1.1009	1.1009	0.334037	0.9395	-1.0644	0.519243	1.5359	1.5359	0.011861	0.7383	-1.3545	0.070189	0.4807	-2.0803	0.013355
B03	*CDK6*	0.5097	-1.9618	0.003065	1.1992	1.1992	0.60039	0.3586	-2.7889	0.001162	0.7774	-1.2863	0.197815	2.1681	2.1681	0.004936
B05	*CDKN1B*	0.6039	-1.6558	0.003151	0.9993	-1.0007	0.891789	0.7431	-1.3457	0.083874	0.4709	-2.1237	0.003767	0.6336	-1.5782	0.03249
B06	*CDKN1C*	0.4448	-2.2484	0.004871	1.6167	1.6167	0.123118	0.6015	-1.6626	0.079657	0.3096	-3.2299	0.01734	0.5148	-1.9427	0.000836
B08	*CDKN2B*	1.3459	1.3459	0.162842	1.5728	1.5728	0.158733	3.3085	3.3085	0.007735	0.4574	-2.1864	0.094492	0.1382	-7.2337	0.034778
B11	*CHUK*	0.8641	-1.1572	0.751892	1.1884	1.1884	0.248637	0.5602	-1.7852	0.000615	1.4538	1.4538	0.003714	2.5954	2.5954	0.000011
B12	*COL1A1*	0.4612	-2.1685	0.695344	1.9735	1.9735	0.301379	2.121	2.121	0.671098	0.0739	-13.5329	0.265298	0.0348	-28.7034	0.019356
C01	*CREB1*	0.4712	-2.1223	0.000013	1.2704	1.2704	0.166243	0.5759	-1.7364	0.00146	0.3704	-2.7001	0.000246	0.6431	-1.555	0.045282
C02	*CREBBP*	0.5733	-1.7442	0.004949	0.9169	-1.0906	0.529271	0.6865	-1.4566	0.085402	0.4618	-2.1652	0.012871	0.6727	-1.4865	0.031713
C05	*EGFR*	1.0733	1.0733	0.576561	1.2094	1.2094	0.592027	1.6854	1.6854	0.100588	0.6246	-1.6011	0.148554	0.3706	-2.6986	0.058977
C06	*EGR1*	0.4064	-2.4606	0.089029	0.866	-1.1547	0.582774	0.5832	-1.7146	0.258818	0.2635	-3.7955	0.203859	0.4518	-2.2136	0.007732
C07	*ELK1*	1.4021	1.4021	0.253766	0.5314	-1.8819	0.118212	1.9408	1.9408	0.035422	0.9491	-1.0536	0.571117	0.489	-2.0448	0.023433
C08	*ETS1*	0.408	-2.4507	0.004651	0.8643	-1.157	0.392341	0.7567	-1.3215	0.200045	0.1945	-5.1424	0.000385	0.257	-3.8913	0.00271
C09	*ETS2*	0.4095	-2.442	0.000021	1.1517	1.1517	0.347857	0.638	-1.5673	0.003854	0.2405	-4.1574	0.000002	0.377	-2.6525	0.00145
C10	*FOS*	0.2186	-4.5752	0.007811	0.6955	-1.4377	0.880356	0.3134	-3.191	0.064792	0.1418	-7.0502	0.049168	0.4526	-2.2094	0.008329
C11	*GRB2*	0.4685	-2.1346	0.000119	0.9897	-1.0104	0.975628	0.7435	-1.345	0.013281	0.2691	-3.7155	0	0.362	-2.7624	0.000907
D01	*HSPA5*	0.3616	-2.7651	0.002835	0.9882	-1.0119	0.744139	0.2563	-3.902	0.009474	0.5467	-1.8291	0.087264	2.1333	2.1333	0.004246
D03	*JUN*	0.1681	-5.9478	0.005626	0.8517	-1.1741	0.94245	0.2684	-3.726	0.052079	0.0959	-10.4256	0.042169	0.3574	-2.7981	0.004825
D05	*KSR1*	0.4104	-2.4366	0.001712	1.0496	1.0496	0.950594	0.3126	-3.1989	0.004865	0.569	-1.7576	0.077619	1.82	1.82	0.01112
D12	*MAP2K6*	0.3917	-2.5529	0.000007	0.6932	-1.4426	0.034078	0.3051	-3.2774	0.000056	0.5286	-1.8917	0.003331	1.7325	1.7325	0.02713
E01	*MAP2K7*	0.6929	-1.4431	0.151298	1.1128	1.1128	0.596204	1.0032	1.0032	0.768644	0.4445	-2.2499	0.000602	0.443	-2.2572	0.019409
E02	*MAP3K1*	0.8643	-1.157	0.835813	1.241	1.241	0.316721	1.6579	1.6579	0.006	0.3956	-2.528	0.004604	0.2386	-4.1912	0.000056
E04	*MAP3K3*	0.5582	-1.7914	0.03076	0.8132	-1.2297	0.479833	0.913	-1.0953	0.529346	0.3093	-3.2328	0.003223	0.3388	-2.9515	0.001123
E05	*MAP3K4*	0.5475	-1.8266	0.004063	1.4293	1.4293	0.400322	0.613	-1.6314	0.042304	0.478	-2.092	0.027125	0.7798	-1.2823	0.365695
E06	*MAP4K1*	0.7349	-1.3607	0.659402	0.5495	-1.8197	0.326671	2.2123	2.2123	0.708124	0.1959	-5.1058	0.263643	0.0885	-11.2954	0.01219
E08	*MAPK10*	0.461	-2.1691	0.000105	1.0285	1.0285	0.922319	0.3498	-2.8585	0.000217	0.6421	-1.5575	0.02503	1.8354	1.8354	0.003841
E11	*MAPK13*	0.8145	-1.2277	0.832559	1.0075	1.0075	0.682454	1.6576	1.6576	0.017713	0.3472	-2.8801	0.016821	0.2095	-4.7739	0.001242
E12	*MAPK14*	0.5163	-1.937	0.001594	1.121	1.121	0.74304	0.7772	-1.2867	0.091153	0.316	-3.1646	0.000189	0.4066	-2.4595	0.00411
F01	*MAPK3*	0.5494	-1.8201	0.000769	1.0596	1.0596	0.917419	0.6494	-1.5399	0.024829	0.4495	-2.2245	0.004195	0.6922	-1.4446	0.057775
F02	*MAPK6*	0.5677	-1.7615	0.002148	1.0301	1.0301	0.9829	0.7283	-1.373	0.058391	0.421	-2.3756	0.004274	0.578	-1.7302	0.022204
F05	*MAPK8IP2*	0.409	-2.4449	0.001979	0.9836	-1.0167	0.601922	0.2964	-3.3742	0.006033	0.602	-1.661	0.078454	2.0314	2.0314	0.014419
F06	*MAPK9*	0.9534	-1.0489	0.97378	1.1697	1.1697	0.48937	0.6889	-1.4516	0.013318	1.408	1.408	0.017683	2.0439	2.0439	0.000257
F09	*MAX*	0.6644	-1.5051	0.006593	1.0507	1.0507	0.761344	0.8917	-1.1214	0.253885	0.4667	-2.1425	0.000068	0.5234	-1.9106	0.007641
F10	*MEF2C*	0.3393	-2.9468	0.000011	1.3261	1.3261	0.19333	0.4775	-2.0944	0.00174	0.2253	-4.4393	0.000095	0.4718	-2.1196	0.043327
F11	*MKNK1*	0.6689	-1.4949	0.109614	0.9945	-1.0056	0.946324	1.0076	1.0076	0.79739	0.4091	-2.4442	0.020769	0.406	-2.4629	0.002534
F12	*MOS*	1.4707	1.4707	0.153155	1.0082	1.0082	0.798707	0.8182	-1.2222	0.076128	2.9726	2.9726	0.017693	3.6332	3.6332	0.092946
G03	*NFATC4*	0.5786	-1.7283	0.725622	1.0264	1.0264	0.677502	1.6051	1.6051	0.141006	0.1701	-5.8797	0.018488	0.106	-9.4375	0.000973
G04	*NRAS*	0.5202	-1.9223	0.000021	1.4019	1.4019	0.028943	0.5825	-1.7168	0.00218	0.4542	-2.2018	0.000612	0.7797	-1.2825	0.059755
G09	*RB1*	0.4957	-2.0171	0.000007	1.2013	1.2013	0.188357	0.5767	-1.7341	0.00112	0.4135	-2.4185	0.000176	0.717	-1.3947	0.031592
G10	*SFN*	2.9153	2.9153	0.01207	1.4456	1.4456	0.625483	32.9166	32.9166	0.000009	0.159	-6.2894	0.100809	0.0048	-207.0238	0.002527

values of p < 0.05 are indicated in red. BT: benign tumor; MT: malignant tumor; G: goiter; PTC: thyroid papillary carcinoma; FTC: thyroid follicular carcinoma.

MAPK pathway genes as potential diagnostic markers in thyroid carcinomas
Virchows Archiv
Breno Pupin[1], Ramon Varella Diniz[1], Maurilio Jose Chagas[2], André Bandiera de Oliveira Santos[3], Renata de Azevedo Canevari[1]*
[1] *Universidade do Vale do Paraíba, UNIVAP, São José dos Campos, SP, Brazil.*
[2] *Hospital Policlin, São José dos Campos, SP, Brazil.*
[3] *Instituto do Cancer do Estado de São Paulo, ICESP, SP, Brazil.*
rcanevari@univap.br

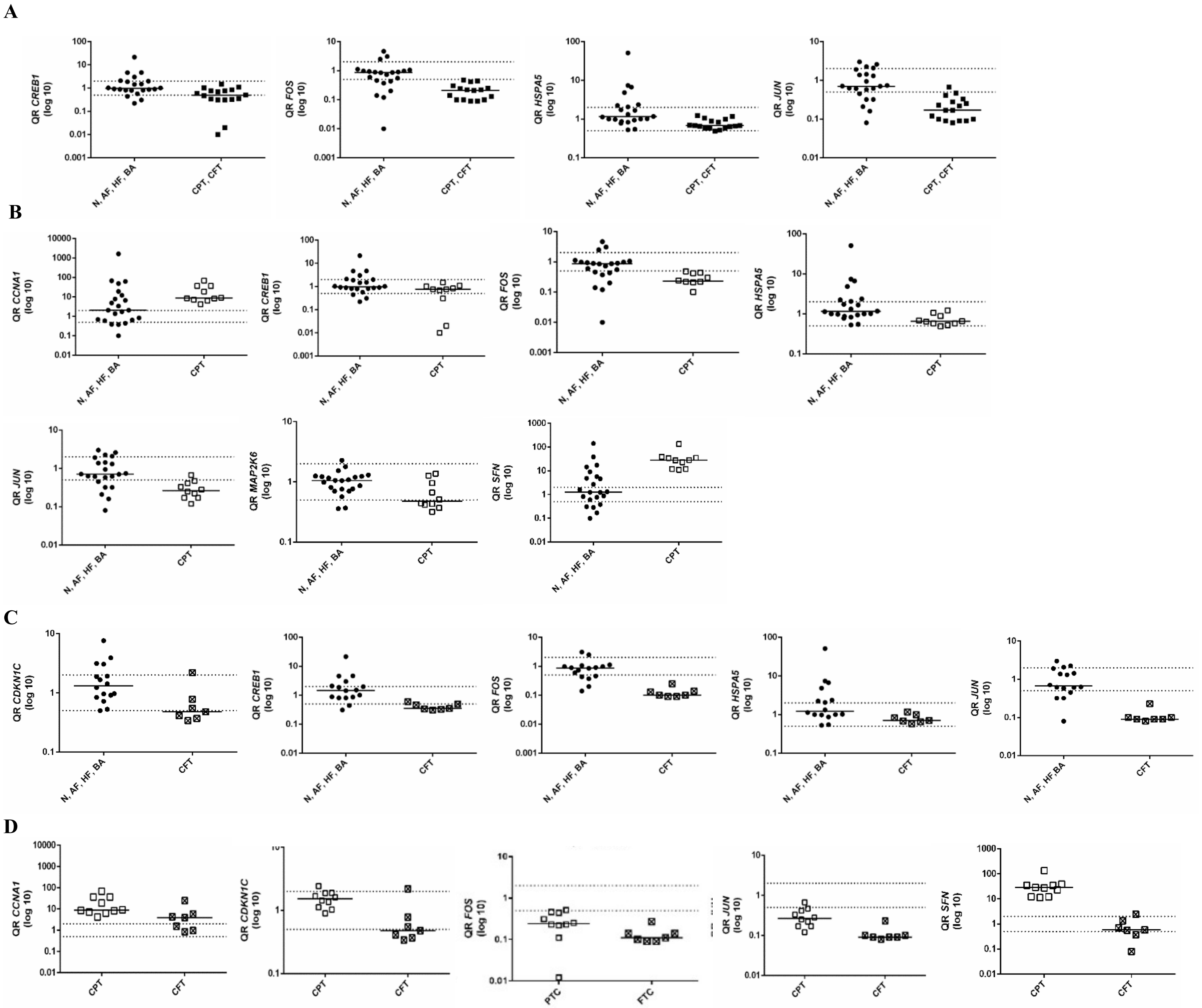

Results obtained by non-parametric Mann Whitney test, with significant values of $P < 0.05$. N: no tumor, BA: adenomatous goiter, AF: follicular adenoma and HF: follicular hyperplasia tissue), CPT: papillary carcinoma and CFT: follicular carcinoma. (A) Analysis 1: differential expression of CREB1, FOS, HSPA5, and JUN genes in the carcinoma samples (PTC and FTC) (group 2) versus normal and benign samples (group 1). (B) Analysis 3: differential expression of CCNA1, CREB1, FOS, HSPA5, JUN, MAP2K6, and SFN genes in the PTC group (group 4) versus group 1. (C) Analysis 4: differential expression of CDKN1C, CREB1, FOS, HSPA5 and JUN genes in the FTC samples (group 5) versus group 1. (D) Analysis 5: differential expression of CCNA1, CDKN1C, FOS, JUN and SFN genes in the FTC samples (group 5) versus PTC samples (group 4).

Suplementary Figure S1. Comparison between the means of expression levels by RT-qPCR of all significantly differentially expressed genes in the studied thyroid samples.
